# Alcoholic hepatitis accelerates early hepatobiliary cancer by increasing stemness and miR-122-mediated HIF-1α activation

**DOI:** 10.1038/srep21340

**Published:** 2016-02-18

**Authors:** Aditya Ambade, Abhishek Satishchandran, Gyongyi Szabo

**Affiliations:** 1 Department of Medicine, University of Massachusetts Medical School, Worcester, MA 01604 United States

## Abstract

Alcohol-related hepatocellular carcinoma (HCC) develops with advanced alcoholic liver disease and liver fibrosis. Using adult mice, we evaluate the effect of alcoholic steatohepatitis on early hepatobiliary carcinoma after initiation by diethyl-nitrosamine (DEN). Here we show that alcohol-fed DEN-injected mice have higher ALT and liver-to-body weight ratio compared to pair-fed DEN-injected mice. Alcohol feeding results in steatohepatitis indicated by increased pro-inflammatory cytokines and fibrotic genes. MRI and liver histology of alcohol+DEN mice shows hepatobiliary cysts, early hepatic neoplasia and increase in serum alpha-fetoprotein. Proliferation makers (BrdU, cyclin D1, p53) and cancer stem cell markers (CD133 and nanog) are significantly up-regulated in livers of alcohol-fed DEN-injected mice compared to controls. In livers with tumors, loss of miR-122 expression with a significant up-regulation of miR-122 target HIF-1α is seen. We conclude that alcoholic steatohepatitis accelerates hepatobiliary tumors with characteristic molecular features of HCC by up-regulating inflammation, cell proliferation, stemness, and miR-122 loss.

Hepatocellular carcinoma (HCC) is the most common liver cancer, and worldwide it represents the fifth most common primary cancer^1^. HCC is also the third leading cause of cancer related mortality globally^2^. In the United States, the incidence of HCC has tripled over the last two decades^3^,^4^. Unlike many other cancers with known associated risk factors, the underlying molecular pathophysiology for HCC is still not completely known. Most commonly, the incidence of HCC is linked to known risk factors including hepatitis B and C, aflatoxin and chronic alcohol consumption^5^. Each of these factors alone poses a significant risk to development of liver cirrhosis^6^. In fact, independent of the initial insult, cirrhosis remains the single common precursor of HCC development.

Hepatocarcinogenesis is a multistep, multistage process that involves genetic and epigenetic alterations that ultimately lead to malignant transformation of hepatocytes^7^. Several animal models have been reported that mimic different steps leading to HCC^8^; however, no animal model of alcohol-related HCC exists that represents human alcoholic liver with features of steatoheptitis and liver fibrosis. Chemically induced HCC models such as the N-nitrosodiethylamine (DEN) induced HCC model are the most widely used and accepted^8^. DEN acts as an alkylating agent for DNA bases which initiates the formation of neoplasms^9^. A recent study combined DEN with alcohol administration in the drinking water^10^; however this form of alcohol feeding fails to result in liver steatosis or inflammation that are characteristics of human alcoholic liver disease^11^. Human HCC is poorly understood, and molecular markers and checkpoints in HCC have recently received attention. It has been shown that the pro-inflammatory cell and cytokine environment, defects in cell proliferation and stem cell-mediated repair all contribute to the multistep process of HCC^8^,^12^,^13^.

In this study, we administered 6 doses of DEN to 4 week old adult C57bl/6 male mice followed by 6 weeks of Lieber-DeCarli alcohol diet. We report a synergistic effect of alcohol with DEN resulting in up-regulation of inflammatory and fibrotic markers and a remarkable induction of early hepatobiliary cancers in mice receiving alcohol-diet and DEN. At the molecular level, livers of alcohol-fed DEN-injected mice show significant up-regulation of markers of cell proliferation, stemness (CD133, nanog), as well as tissue inflammation and up-regulation of hypoxia inducible factor-1α and its target, VEGFR1. These results indicate that chronic alcohol accelerates hepatobiliary cancer via multiple key mechanisms in carcinogenesis.

## Results

### Combination of alcohol and DEN induces sustained liver injury

**Figure 1:**
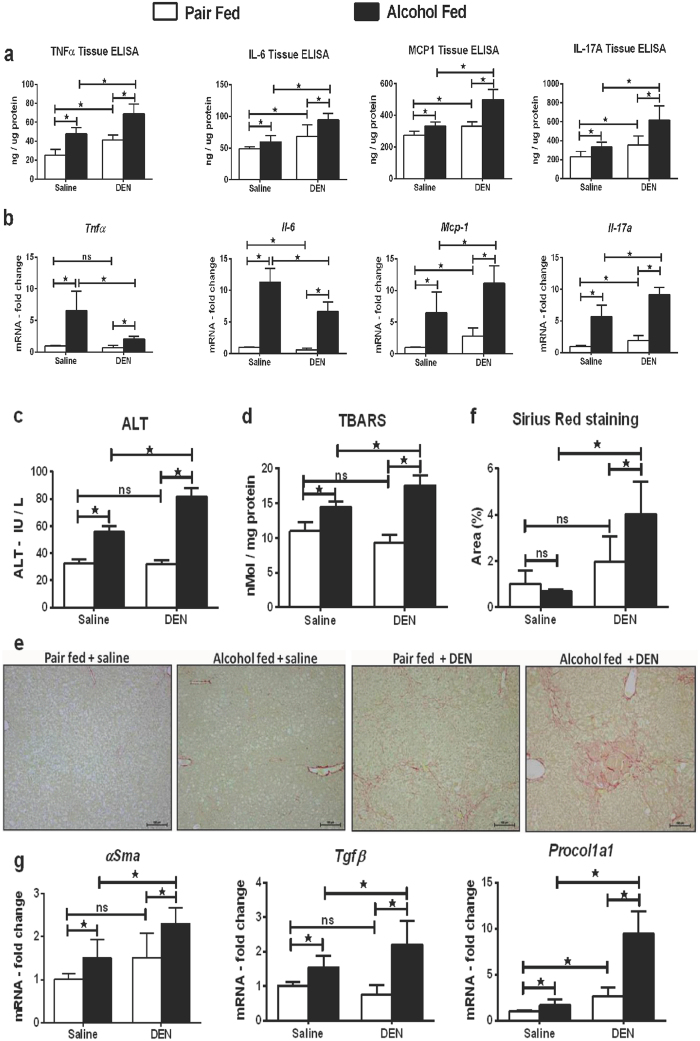
Alcohol and DEN are additive in induction of liver inflammation and fibrosis. (**a**) Tissue protein levels of pro-inflammatory cytokines (n ≥ 5mice per group). (**b**) Fold changes in mRNA levels of pro-inflammatory cytokines in liver tissue. (**c**) Serum ALT levels at sacrifice. (**d**) Liver tissue ROS measured by TBARS assay. (**e**) Representative Sirius Red staining images from all experimental groups. Bars inside the images indicate 100 μm. (**f**) For quantification, at least 3 different microscope fields at 10x magnification were scored for each mouse (n ≥ 5mice per group). Bar graph shows percent Sirius red positive area quantified using ImageJ. (**g**) Fold changes in mRNA levels of fibrosis markers in liver tissue. In all graphs, values are given as average ± SD, ANOVA and Dunnett’s multiple comparison were used to compare the means of multiple groups; (*p < 0.05).

Chronic alcohol use is a major risk factor for HCC development in humans^4^. Here we hypothesized that the alcoholic liver environment accelerates liver tumor development after repeated administration of a chemical carcinogen, DEN. In this study, male C57bl/6 mice received 6 weekly DEN injections, starting at age of 4 weeks followed by chronic alcohol administration (Supplementary Fig. S1). Chronic alcohol exposure induces inflammation that is driven by secretion of pro-inflammatory cytokines^14^. We analyzed TNFα, MCP-1, and IL-6 expression at the protein and mRNA levels in the liver tissue and observed that expression of these pro-inflammatory cytokines at the protein level was significantly higher in alcohol-fed DEN-injected mice as compared to pair-fed DEN-injected or alcohol-fed saline-injected mice (Fig. 1a). Interestingly, the expression of TNFα and IL-6 at the mRNA level in alcohol-fed DEN-injected mice was lower than alcohol-fed saline-injected mice (Fig. 1b). IL-17A is an inflammatory and immunoregulatory cytokine secreted by Th17 cells, γδ T cells and has been recently reported to promote tumor growth in hepatocellular carcinoma^15^. While both alcohol and DEN, respectively increased IL-17A levels in our experimental model, IL-17A induction was additively increased by combination of DEN and alcohol in the liver at the tissue protein (Fig. 1a) and mRNA (Fig. 1b) levels, compared to pair-fed DEN-injected and alcohol-fed saline-injected mice. Serum ALT was significantly higher in alcohol-fed DEN-injected mice compared to any other group (Fig. 1c). Liver to body weight ratio at sacrifice was significantly higher in alcohol-fed DEN-injected mice as compared to alcohol-fed saline-injected and pair-fed DEN-injected mice (Supplementary Fig. S2). Alcohol induced oxidative stress as measured by thiobarbituric acid reactive substances (TBARS) was significantly higher in alcohol-fed DEN-injected mice compared to other experimental groups (Fig. 1d).

HCC in human alcoholics develops in fibrotic and cirrhotic livers^16^. The Sirius Red staining suggested a higher degree of fibrosis in alcohol-fed DEN-injected mice compared to other groups (Fig. 1e,f). To estimate the extent of fibrosis, we evaluated α-SMA, procollagen1α and TGFβ expression in the liver. As shown in Fig. 1g, all markers of fibrosis were significantly up-regulated in alcohol-fed DEN-injected mice (Fig. 1g). These data indicated significant inflammation and fibrosis in our experimental model.

### Alcohol accelerates HCC development after DEN insult

**Figure 2:**
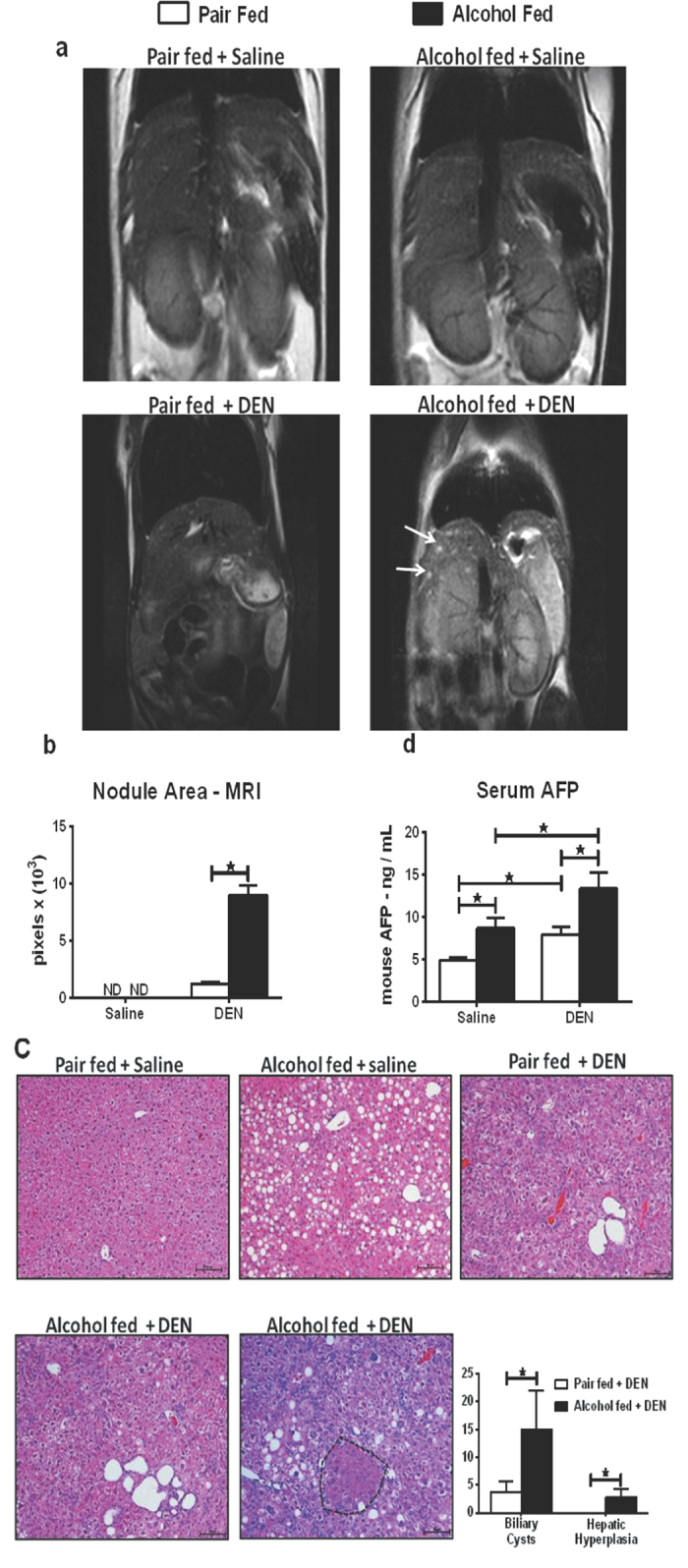
Alcohol accelerates HCC development after DEN insult. (**a**) T2-weighted MRI of liver in coronal section. Arrows denote cysts. (**b**) Quantification of cyst area using ImageJ. (**c**) Representative H & E stained liver sections from all treatment groups. Bars inside the images indicate 100 μm. Biliary cysts were observed in both DEN-injected groups while the hepatic hyperplasia (encircled in black dotted line) was exclusively observed in alcohol + DEN mice. Bar graph shows average number of biliary cysts and hepatic hyperplastic nodules counted at 10× magnification for each mouse (n ≥ 5 mice per group). (**d**) Fold changes in serum AFP levels. In all graphs, values are given as average ± SD, ANOVA and Dunnett’s multiple comparison were used to compare the means of multiple groups; (*p < 0.05).

Having confirmed the inflammation status, fibrotic injury in our samples, next we assessed HCC markers using 3 complementary strategies: MRI, histology and serum markers. First, the T2 weighted MRI scan of alcohol-fed DEN-injected mice showed significantly higher number of characteristic intrahepatic biliary cysts visualized as bright spots while the pair-fed DEN-injected mice had very few cysts (Fig. 2a). Pixel quantification of these MRI scans using ImageJ revealed significantly higher number of nodules in alcohol-fed DEN-injected mice compared to the pair-fed plus DEN group (Fig. 2b). Nodules were not found in pair-fed saline-injected and alcohol-fed saline-injected mice.

Second, histopathology examination of the liver sections independently confirmed the presence of intrahepatic biliary cysts and hepatic hyperplastic nodules in alcohol-fed DEN-injected mice (Fig. 2c). In addition to higher number of intrahepatic biliary cysts, the alcohol-fed DEN-injected mice exclusively showed hepatic hyperplastic nodules pointing towards the role of alcohol as a tumor promoter. Quantification of the cysts and nodules in liver sections revealed significantly higher number of cysts and nodules, respectively, in livers after chronic alcohol plus DEN injection compared to any other experimental groups (Fig. 2c).

Third, serum AFP levels were significantly higher in alcohol-fed DEN-injected mice as compared to pair-fed DEN-injected and alcohol-fed saline-injected mice (Fig. 2d). Taken together, the MRI, histopathology, and the serum AFP data provided evidence for increased number of biliary cysts and accelerated development of hepatic hyperplasia in mice receiving alcohol plus DEN.

### Chronic alcohol induces stemness

**Figure 3:**
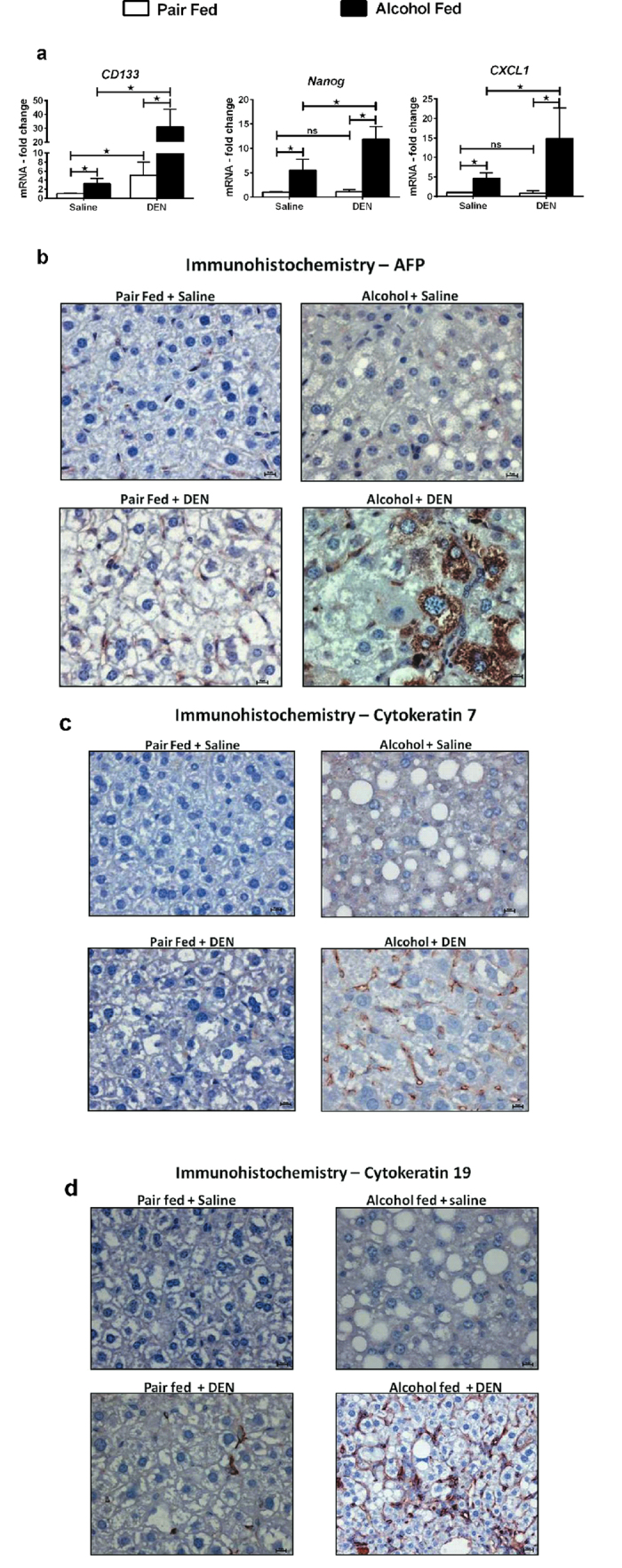
Chronic alcohol induces stemness. (**a**) Fold changes in mRNA levels of stem cell markers in liver tissue (n ≥ 5 mice per group). (**b**) Representative immunostaining images for AFP showing more positive cells in alcohol + DEN mice, (**c**) CK7 and (**d**) CK19, respectively. Bars in (**b–d**) indicate 10 μm. In all graphs in panel (**a**) values are given as average ± SD, ANOVA and Dunnett’s multiple comparison were used to compare the means of multiple groups; (*p < 0.05).

Two recent independent studies suggested that cancer growth is mediated by a small population of stem-like cells, referred to as cancer stem cells (CSCs) or tumor initiating cells (TICs) that are characterized by expression of two stem cell markers, CD133 and nanog^17^,^18^. In our model, expression of CD133 and nanog was significantly higher in alcohol-fed DEN-injected mice compared to pair-fed DEN-injected or alcohol-fed saline-injected mice (Fig. 3a). CD133^+^ liver tumor initiating cells have been shown to promote tumor growth via IL-8/CXCL1 signaling in humans^17^. Expression of CXCL1 was significantly higher in alcohol-fed DEN-injected mice suggesting that CXCL1 signaling may be involved in promoting tumor growth in this experimental model (Fig. 3a).

The expression of AFP is directly associated with hepatocyte differentiation^19^. Immunohistochemistry staining showed an abundance of AFP positive cells in alcohol-fed DEN-injected mice compared to all other groups (Fig. 3b). Bipotent progenitor cells or stem cells within the liver, termed as oval cells, are implicated in the pathogenesis of hepatocellular carcinoma and cholangiocarcinoma in animal models and may be important in the development of hepatocellular carcinoma in human chronic liver diseases^20^. In addition, oval cells can also directly de-differentiate from mature liver cells such as hepatocytes^21^. These oval cells have been reported to express dual lineage markers, such as alphafetoprotein (AFP) as hepatocyte marker^22^ and cytokeratins 7 and 19 as markers of bile duct epithelium^23^. Immunostaining for CK7 and CK19 revealed increased number of these markers of biliary progenitors (CK7 positive cells) and hepatic stem cells or oval cells (CK19 positive cells) in the livers of alcohol-fed DEN-injected mice, respectively (Fig. 3c,d).

### Chronic alcohol up-regulates hepatocyte proliferation and Epithelial Mesenchymal Transition (EMT)

**Figure 4:**
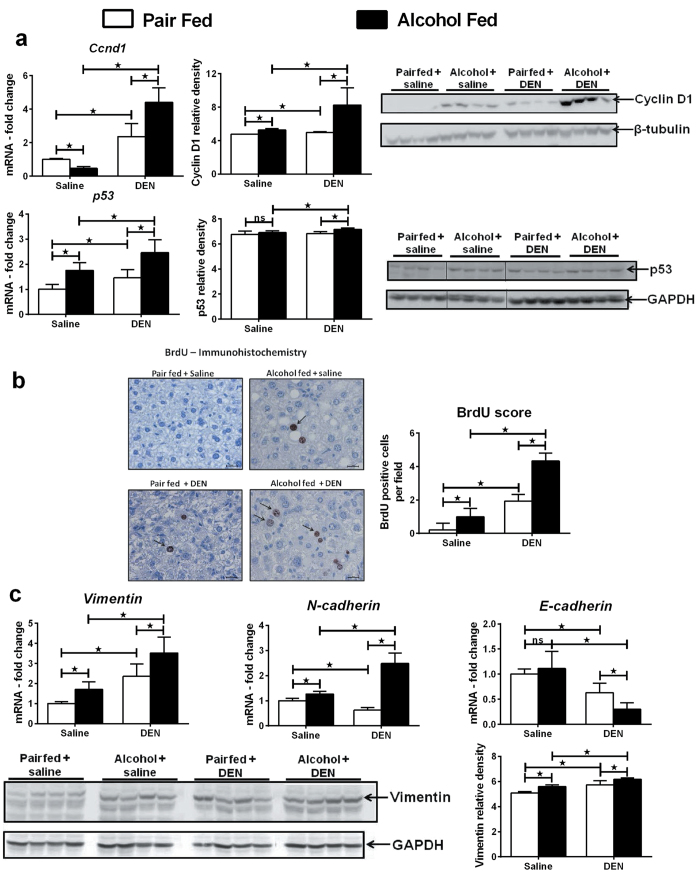
Chronic alcohol up-regulates proliferation, EMT markers. (**a**) Liver mRNA and protein levels of cyclin D1 and p53. β-tubulin and GAPDH used as loading controls for western blots. Relative density of signal is shown for each protein blot. (**b**) Representative immunostaining images for BrdU incorporation. Arrows point to positive staining. BrdU positive nuclei were scored in at least 3 different microscopic fields for each mouse. Bars indicate 10 μm. (n ≥ 5 mice per group). (**c**) Liver mRNA and protein levels of vimentin with relative density of vimentin protein. GAPDH used as loading control for western blots. Liver mRNA levels of N-cadherin and E-cadherin. In all graphs, values are given as average ± SD, ANOVA and Dunnett’s multiple comparison were used to compare the means of multiple groups; (*p < 0.05).

First, we evaluated the expression of cyclin D1 and p53, signature molecules implicated in HCC progression^24^. As shown in Fig. 4a, the expression of both cyclin D1 and p53 was significantly higher in alcohol-fed DEN-injected mice at the mRNA and protein levels compared to all other groups (Fig. 4a). To provide evidence of cell proliferation, we injected the mice with bromodeoxyuridine (BrdU), the S phase marker before sacrifice. As shown in Fig. 4b, higher number of cells was stained BrdU positive in alcohol-fed DEN-injected mice (Fig. 4b).

HCC development is characterized by epithelial mesenchymal transition (EMT)^25^. Consistent with this, the epithelial mesenchymal transition markers, N-cadherin and vimentin, were significantly up-regulated at the mRNA level in alcohol-fed DEN-injected mice (Fig. 4c). Up regulation of vimentin in alcohol-fed DEN-injected mice was also seen at the protein level (Fig. 4c). Loss of E-cadherin has been shown to promote liver carcinogenesis^26^. The expression of E-cadherin was significantly reduced in alcohol-fed DEN-injected mice as compared to alcohol-fed saline-injected and pair-fed DEN-injected mice (Fig. 4c).

### Chronic alcohol induces hedgehog signaling

**Figure 5:**
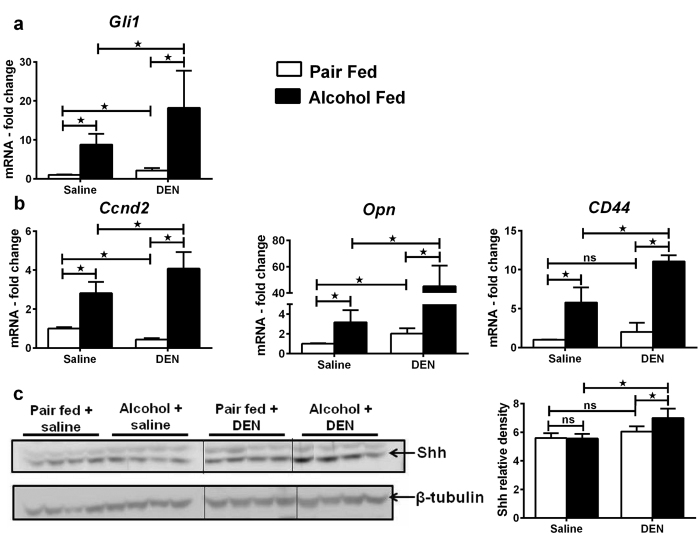
Up-regulation of hedgehog signaling in experimental HCC model. (**a**) Liver mRNA levels of Gli-1. (**b**) Liver mRNA levels of hedgehog target genes, Ccnd2 (cyclinD2), Opn (osteopontin) and CD44. (**c**) Liver protein levels of Shh, the ligand for hedgehog pathway with relative band intensity. In all graphs, values are given as average ± SD, ANOVA and Dunnett’s multiple comparison were used to compare the means of multiple groups; (*p < 0.05).

Progression of hepatobilliary cancer involves activation of multiple intracellular regulatory pathways^7^. We analyzed the hedgehog (Hh) signaling, a major signaling pathway reported to be dysregulated in HCC^27^. Upon activation of the surface receptor PTCH1/2 by Hh ligands, downstream transcription factors Gli1/2/3 are activated which drive the expression of several Hh target genes including Cyclin D2, OPN and CD44. The expression of Gli1 was significantly higher in alcohol-fed DEN-injected mice as compared to alcohol-fed saline-injected or pair-fed DEN-injected mice (Fig. 5a). Hh targets, CCND2, OPN and CD44 were significantly up-regulated affirming the activation of Hh pathway in this model (Fig. 5b). Sonic hedgehog (Shh), the ligand for Hh pathway showed significant protein induction in liver from alcohol-fed DEN-injected mice (Fig. 5c). These data suggest that alcohol alters the expression of Hh target genes and dysregulates Hh signaling contributing to progression of HCC.

### Altered liver microRNA-122 and HIF-1α correlates with HCC in mice

**Figure 6:**
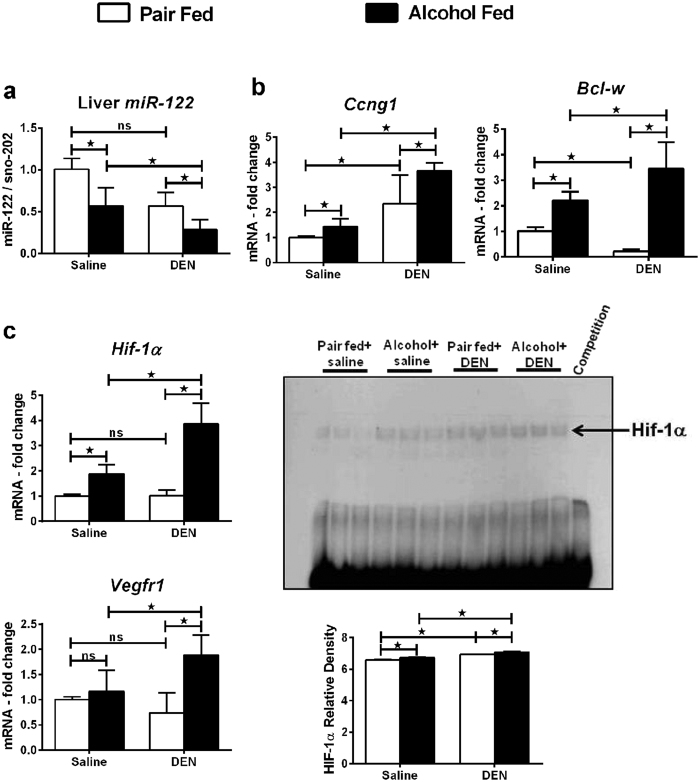
Altered microRNA-122 and HIF-1α expression in liver confirms HCC in mice. (**a**) miR-122 levels in liver. (**b**) Targets of miR-122, Ccng1 (cyclinG1) and Bcl-w mRNA in liver. (**c**) Liver HIF-1α mRNA levels, HIF-1α DNA binding activity assayed by EMSA and target gene Vegfr1 mRNA levels (n ≥ 5 mice per group). In all graphs, values are given as average ± SD, ANOVA and Dunnett’s multiple comparison were used to compare the means of multiple groups; (*p < 0.05).

MicroRNAs are small non-coding RNA molecules that regulate post transcriptional gene expression via RNA silencing. miRs have been reported to control the liver tumor development and aggressiveness^28^. The most abundant miRNA in the liver is miR-122. Decreases in liver tissue miR-122 have been correlated with gain of metastatic properties of liver cancer and increased mortality^29^. We found that the expression of miR-122 in the liver tissue was significantly lower in alcohol-fed DEN-injected mice compared to any other groups in this study (Fig. 6a). miR-122 regulates the expression of cyclin G1, whose high levels have been reported in several human cancers^30^. In addition, by modulating cyclin G1, miR-122 influences p53 protein stability and transcriptional activity and reduces invasion capability of HCC-derived cell lines^31^. We observed an up-regulation of cyclin G1 (Fig. 6b) and p53 (Fig. 4a) in the liver. Bcl-w, an anti-apoptotic gene and a target of miR-122, was significantly up-regulated in alcohol-fed DEN-injected mice (Fig. 6b).

Tumor tissues are often characterized by low levels of tissue oxygen known as hypoxia, which makes tumor tissues resistant to radiotherapy^32^. Hypoxia induces the transcription factor HIF-1α which drives the expression of various cell proliferation and angiogenesis genes^33^. We recently discovered that HIF-1α is a miR-122 target^34^. Indeed, alcohol-fed DEN-injected mice showed an up-regulation in HIF-1α mRNA and a remarkable increase in its DNA binding activity compared all other groups (Fig. 6c). The expression of VEGFR1, a HIF-1α target gene which plays pivotal a role in angiogenesis, was also significantly higher in alcohol-fed DEN-injected mice indicating an up regulation in the biological activity of HIF-1α (Fig. 6c).

### Serum miR-122 increase correlates with liver injury and tumor markers

**Figure 7:**
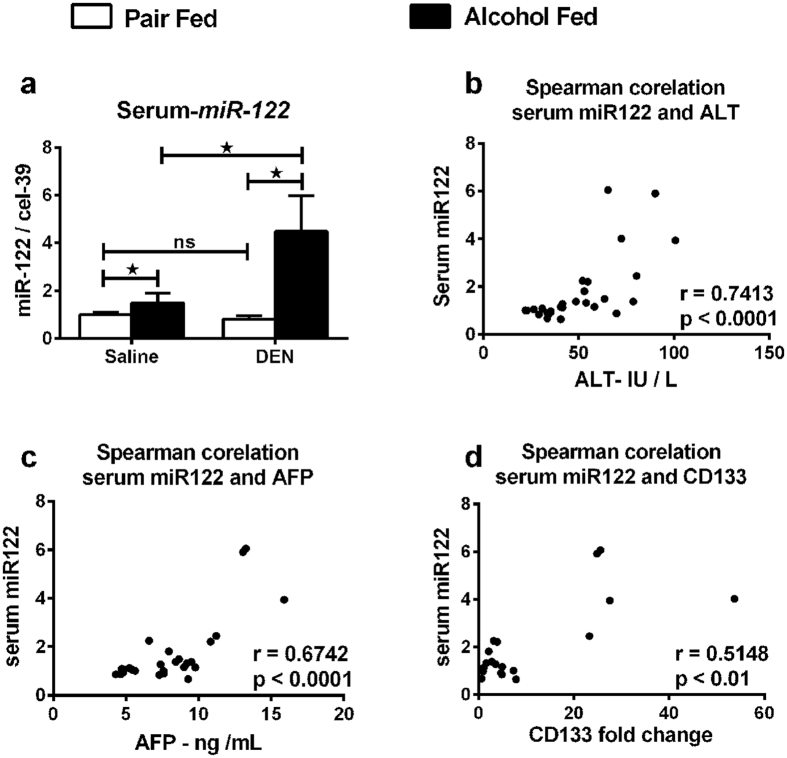
Up-regulation of serum miR-122 co-relates with liver injury markers. (**a**) Serum miR-122 levels. (**b**–**d**) Spearman co-relation of serum miR-122 with serum ALT, serum AFP and liver CD133 respectively. In all graphs, values are given as average ± SD, ANOVA and Dunnett’s multiple comparison were used to compare the means of multiple groups; (*p < 0.05).

Circulating microRNAs have a potential to serve as biomarkers of disease^35^. Levels for circulating miR-122 can be useful in predicting liver diseases such as HCC and ongoing liver injury^36^. Hence, we analyzed expression of miR-122 in serum collected at sacrifice from mice. Serum miR-122 levels were highly increased in alcohol-fed DEN-injected mice compared to all other groups (Fig. 7a) and showed a positive correlation with ALT increase (Fig. 7b) and serum AFP (Fig. 7c). Lastly, a significant correlation was seen between serum miR-122 and CD133 expression (Fig. 7d). Taken together, our data provides experimental evidence for alcohol accelerating DEN initiated development of HCC in mice.

## Discussion

**Figure 8:**
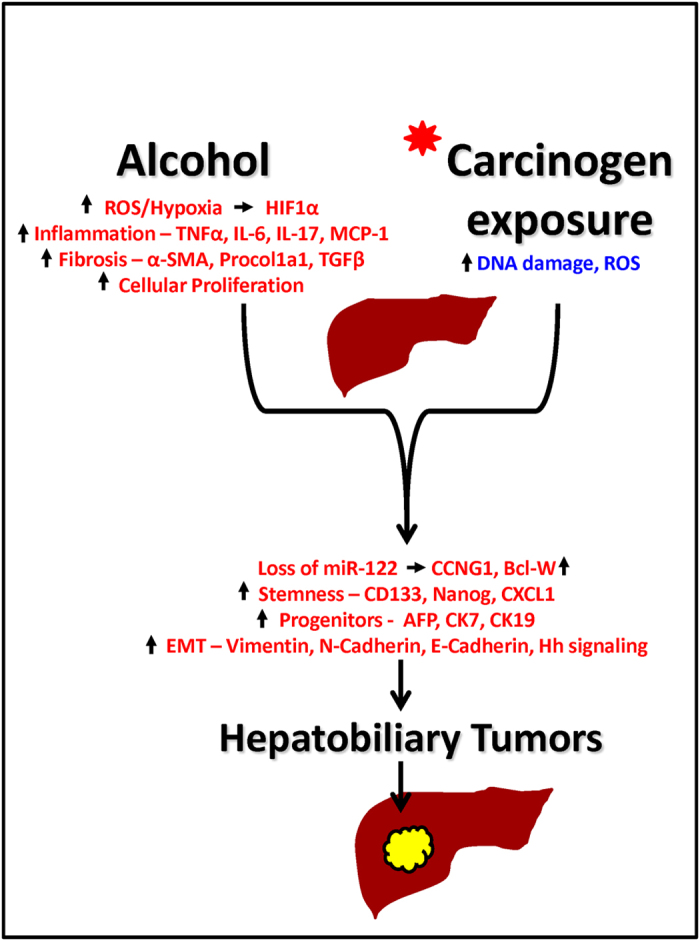
Proposed pathogenic model for alcoholic hepatitis accelerated hepatobiliary cancer. Chronic alcohol exposure leads to increased ROS, inflammation and fibrosis which leads to cellular hypoxia and proliferation and accelerates the carcinogen induced progression of hepatobiliary cancer. The hepatobiliary cancer is characterized by loss of miR-122 and up regulation of its targets cyclinG1 and Bcl-W, increased stemness, progenitor cell markers and enhanced EMT.

Chronic alcohol use by itself leads to fatty liver, liver inflammation, and cirrhosis^37^. Epidemiological data suggests that chronic heavy alcohol consumption is a significant risk factor towards the development of HCC^4^. In this study we demonstrate that chronic alcohol feeding in adult mice accelerated DEN (chemical) induced liver tumor development with molecular characteristics of HCC. We found that features of human alcoholic hepatitis; inflammation and fibrosis were increased in DEN-injected mice after alcohol feeding and resulted in increased numbers of biliary cysts and early HCC. Our experiments dissected molecular mechanisms involved in early hepatic carcinogenesis and found that markers of stemness (CD133 and nanog), factors involved in epithelial mesenchymal transition (vimentin and hedgehog activation) and miR-122 decrease, were all present in livers with early liver tumors triggered by alcohol plus DEN. Our data indicate that increased progenitor cell activation is triggered in the alcoholic liver tissue microenvironment that is characterized by high levels of pro-inflammatory signals from both innate (TNFα and MCP-1), adaptive immunity (Th17) and activation of HIF-1α. In addition to steatohepatitis induced by chronic alcohol administration, liver fibrosis was also present as indicated by Sirius Red staining, alpha-smooth muscle actin and collagen deposition in the liver (Fig. 8).

Although several carcinogen initiated HCC animal models have been described, none of them employed alcohol as a dietary component or tumor promoting agent^8^. A recent study performed in neonate mice combined chronic alcohol exposure in the drinking water with DEN injection and showed tumors at 48 weeks^10^. However, alcohol administration in the drinking water does not cause alcoholic liver disease^11^. In our current study, we employed the Lieber-DeCarli alcohol feeding model that results in features of human alcoholic liver disease including steatosis, inflammation and liver fibrosis^38^,^39^. By utilizing adult mice and the Lieber-DeCarli diet, our model displays the natural course of progression of alcoholic liver disease as well as hyperplastic changes as shown by histology and modulation of molecular markers suggestive of early hepatic tumors in a short time (12 weeks). However, because of the relatively short alcohol administration, our model cannot provide a direct evidence of alcohol increasing liver cancer and a longer follow up will be required to overcome this limitation. Nonetheless, our model resembles the human condition of carcinogen pre-exposure followed by long term alcohol consumption with features of human alcoholic liver disease and liver fibrosis.

DEN is metabolized in the liver by Cyp2E1, the same enzyme that metabolizes alcohol^40^. It has been proposed that the charged nucleophilic intermediates of DEN attack DNA bases thereby leading to mutations which initiate cellular transformation^9^. Alcohol metabolites also generate ROS, cause inflammation and steatosis. The combined effect of these two can lead to hepatocyte transformation (Fig. 8). Because both DEN and alcohol are primarily metabolized in hepatocytes, the chronic alcoholic injury may accelerate hepatocyte transformation leading to appearance of early neoplastic foci found in alcohol plus DEN mice only. We confirmed increased reactive oxygen species (ROS) in alcohol plus DEN mice as indicated by elevated TBARS. Chemical carcinogens exert their effect by generating ROS during their metabolism in the liver, which is responsible for DNA damage associated with increased risk of cancer development^41^. Sustained increased ROS can activate a variety of transcription factors including NF-κB, p53, HIF-1α, β-catenin/Wnt, and Nrf2 in liver leading to chronic inflammation that is responsible for cancer progression^42^. Our data suggests that in this experimental model, hepatocytes from alcohol plus DEN mice are exposed to higher levels of ROS than pair-fed DEN-injected hepatocytes, which may contribute to their accelerated transformation to tumors.

DEN induced cancer models in animals show the occurrence of biliary cysts, which has been attributed to oxidative stress in the liver^43^. Biliary cysts are known precursors for cholangiocarcinoma (CCA)^44^. Cholangiocarcinoma is the second most common primary hepatic malignancy with 5-year survival rate below 5%^45^. Further, alcoholic liver disease has been shown to be an established risk factor for CCA^46^. Biliary cysts were observed in all DEN-injected groups in our experimental model. However, mice receiving both alcohol and DEN showed both hepatic hyperplasia and biliary cysts. Cellular transformation of hepatic and biliary progenitors points towards the role of alcohol as tumor promoting agent for both hepatocyte and cholangiocyte (biliary epithelial) cell lineages.

The well accepted theory in cancer biology states that cancer originates from a small number of cancer stem cells (CSCs) or tumor initiating cells (TICs)^47^. These cells express unique stem cell markers which are not expressed by their adult differentiated counterparts. This small population of CSCs is able to extract more nutrients from the blood stream and proliferate faster^48^. We report significant up-regulation in AFP and CK19 both, markers of oval cells which are bipotent precursor cells in the liver that can contribute to the formation of hepatocytes as well as bile ducts^22^,^23^ and have been implicated in development of HCC and CCA^20^. However, recently it was shown that oval cells can also de-differentiate directly from mature liver cells such as hepatocytes^21^. Hence, the precise origin of these oval cells in our experimental model remains uncertain. A time course or *in vivo* cell lineage tracing study will help to ascertain the origin of these oval cells. Interestingly, adult murine liver oval cell population has been shown to express CD133, another stem cell marker^49^. CD133 and Nanog have been useful in identifying this CSC population in HCC^17^,^18^. The significant increase in liver CD133 and nanog of alcohol-fed DEN-injected mice suggests a synergistic effect of alcohol and DEN on the HCC cancer stem cells. Further, the CD133/CXCL1 pathway is shown to be up-regulated in human HCC^17^, whereas TGFβ can induce EMT transition and stem cell properties in CCA^50^. Given the highly elevated CXCL1 and TGFβ in alcohol plus DEN mice, a combination of both these may accelerate development of hepatobiliary cancer in our model. The presence of multiple well established stem cell makers (CD133, nanog, AFP, CK7, and CK19) provides strong evidence to the involvement of oval cells in development of HCC in this model. DEN pre-exposure could selectively up-regulate CK19 in oval cells and alcohol may accelerate the proliferation of these cells to the mixed phenotype of hepatobiliary cancer seen in our model. Tumor tissue is often characterized by up-regulation of proliferation markers. In addition, tumor tissue overexpresses EMT markers which are useful in hyperproliferation and angiogenesis. Loss of E-cadherin and up-regulation of N-cadherin along with induction of CCND1 are direct indicators of hyperproliferative hepatobiliary cells in alcohol plus DEN mice^26^.

Additional evidence of accelerated HCC development by alcohol is up-regulation of the Hedgehog (Hh) pathway in our model. Hh plays an important role in the development of tissues and is highly active during early stages of life^27^.The transcription factors associated with Hh signaling control the expression of several proteins important in cell cycle, cell differentiation and maturation^27^. Treatment with Hh antagonist is reported to promote regression of HCC in murine model^51^. Upon binding of Hh ligand to its receptor PTCH1, a seven membrane spanning receptor Smoothened (Smo) activates the downstream transcriptional cascade via Gli proteins. Using purmorphamine, an agonist of Smo, Gores *et al*. discovered the role of non-canonical Hh signaling in CCA^52^. The up-regulation of Gli-1, Hh target genes and the ligand Shh in alcohol plus DEN mice suggest that Hh pathway may drive the progression of the mixed phenotype of HCC and CCA in our experimental model.

The most abundant miRNA in the liver is miR-122. miR-122 expression increases during embryogenesis until it constitutes 72% of total miRNA in adult human liver^53^. However, decrease in hepatic miR-122 is known to be a tumor-specific event in humans^54^ as well as experimental animal models^53^. Anti-inflammatory and anti-tumorigenic role for hepatic miR-122 has been reported^55^. Further, loss of tissue miR-122 expression in liver cancer has been reported to correlate with gain of metastatic properties^29^. Consistent with this, we found decreased liver miR-122 and increased expression of molecular markers of HCC in DEN plus alcohol treated mice. Overexpression of miR-122 reduces tumorigenic properties in HCC cell lines^56^ and recent reports propose a unique therapeutic potential for miR-122 in liver diseases^57^. A number of validated miR-122 targets including cyclin G1, ADAM10, IGF1R, SRF, ADAM17 and Wnt1 are shown to be involved in hepatocarcinogenesis, epithelial-mesenchymal transition, and angiogenesis^31^,^56^,^58^. Our lab has recently shown that microRNA-122 regulates HIF-1α in hepatocytes in a diet-induced steatohepatitis model^34^. Hypoxia induced HIF-1α, a well studied transcription factor in cancer models, plays a pivotal role in advancement of tumor^59^. Interestingly, chronic alcohol is also known to induce HIF-1α expression and activity in the liver^60^. Thus, the hepatic HIF-1α activity may be under dual regulation in our model. Up-regulation of HIF-1α DNA binding activity and VEGFR1 mRNA in our experimental model upholds a role for miR-122 regulated HIF-1α in HCC development and progression.

Circulating miRs have been reported for their potential as biomarkers of drug induced liver injury^35^ as well as NASH^61^. Recent reports found that the change in plasma miR-122 concentration precedes the increase in aminotransferase activity in the blood, making it one of the earliest markers of liver injury^62^. Circulating miR-122 has been reported as a novel potential biomarker for diagnosis of different types of liver diseases including HCC^35^,^57^,^63^. Serum miR-122 was elevated in patients with HCC or chronic hepatitis^64^. A correlation of serum miR-122 with clinical chemistry parameters of liver injury, hepatic necro-inflammation is known and therefore use of serum miR-122 levels as prognostic markers in patients with hepatocellular carcinoma is suggested^36^. Our experimental data here shows a significant increase in serum miR-122 with a concurrent loss of miR-122 in the liver tissue thus acknowledging the proposed the role for miR-122 as a HCC biomarker in animal models. We found a significant positive correlation of serum miR-122 levels with established clinical markers of liver injury, ALT, serum AFP and histology.

In summary, by combining multiple DEN injections prior to chronic Lieber-DeCarli alcohol diet feeding, we have developed a mouse model that displays the features of hepatobiliary tumor. Our model involves a sequential step-wise progression of alcoholic liver disease to hepatobililary cancer with molecular signatures of HCC (Fig. 8). Importantly, our model involves mice with alcoholic steatohepatitis and has all the features usually associated with pathogenesis and diagnosis of human HCC. This combination presents one of the most unique phenomenons of chronic alcohol leading to progression of HCC that occurs in humans. In our opinion, availability of such a model will enable testing anti-cancer drugs in preclinical setup as well as provide opportunities to understand alcohol associated molecular and cellular mechanisms related to HCC.

## Methods

### Animal model of hepatobiliary carcinoma

To establish a mouse model of hepatobiliary cancer based on Lieber-DeCarli alcohol diet, we injected 4 week old C57bl/6 male mice with total 6 doses of DEN (Sigma, St. Louise, MO) intraperitoneally. As shown in supplementary Fig. S1, a dose of 75 mg/kg DEN was administered weekly for first 3 weeks and for later 3 weeks a dose of 100 mg/kg DEN was injected i.p. At week 8, the mice were divided into alcohol and pair-fed (control) groups. Two age matched groups of mice without DEN were included in the study to understand the effect of chronic alcohol feeding (Supplementary Fig. S1). Depending on the experimental design, mice were fed 4% Lieber-Decarli alcohol diet or calorie matched control diet for 6 weeks. At sacrifice, blood and liver tissues were collected for further assays. The study protocol was approved by the Institutional Animal Use and Care Committee of the University of Massachusetts Medical School. All the methods were carried out in accordance with the approved guidelines.

### Magnetic Resonance Imaging

Magnetic resonance imaging (MRI) of liver was performed to monitor hyperplastic changes in liver. Images were obtained using 3T Philips Achieva whole-body MR scanner (Philips Medical Systems, Best, Netherlands) with a custom-made solenoid T/R coil with a diameter of 30 mm. The animals were anesthetized with 5% isoflurane mixed with carbogen (95% O_2_/5% CO_2_) and were maintained with 1% to 2% isofluorane. Coronal T2–weighted spin echo images were acquired with respiratory triggering to reduce the motion artifacts. The respiration rate was monitored with an optical probe (Model 1025 T Monitoring and Gating System, SA Instruments Inc, Stony Brook, NY). The output signal from the respiration monitor was used to trigger, in real time, the MR acquisition. As a consequence of the triggered acquisition, the TR value of around 2000 ms, corresponding to the respiration rate of around 30 bpm, was determined. Other imaging parameters were: echo time (TE) of 70 ms, flip angel of 90 degrees, TSE-factor of 8, number of average = 4, matrix size of 148 × 120, field of view of 30 × 25 mm^2^, slice thickness of 1 mm with no gap, acquisition time around 4 mins for 22 slices. Hyperplastic nodules were distinguished from normal liver tissues on basis of differences in homogeneity and signal intensity. Pixel based nodule area quantitation was performed using ImageJ software.

### Biochemical Assays and cytokines

Serum alanine aminotransferase (ALT) activity was determined using a kinetic method (TECO Diagnostics, Anaheim, CA). Intracellular cytokine levels were monitored in liver whole cell lystate using TNFα, IL-6, MCP-1 ELISA kits (Biolegend, San Diego, CA). Liver thiobarbituric acid reactive substances (TBARS), a measure of oxidative stress, were estimated using TBARS assay kit (ZeptoMetrix, Buffalo, NY). Tissue IL-17 and serum AFP were assayed by ELISA (R&D systems, Minneapolis, MN).

### EMSA

The DNA binding activity of HIF-1α was assessed by electrophoretic mobility shift assay as described previously^60^. Briefly, nuclear protein extract from liver (5 μg) was incubated with 50,000 cpm γ ^32^P-labeled HIF-1α consensus oligonucleotide at room temperature for 30 min. All reactions were run on a 4% polyacrylamide gel, and the dried gel was exposed to an X-ray film at −80 °C for different times. For the cold competition reaction, a 20-fold excess of same, unlabeled, double-stranded oligonucleotide was added to the reaction mixture before adding the labeled oligonucleotide probe.

### RNA extraction and real-time PCR

Total RNA was extracted using the Direct-zol RNA MiniPrep according to the manufacturer’s instructions (Zymo Research, Irvin, CA). RNA was quantified using Nanodrop 2000 (Thermo Scientific, Wilmington, DE). Complementary DNA (cDNA) synthesis was performed by reverse transcription of total RNA using the iScript Reverse Transcription Supermix (BIO-RAD, Hercules, CA). Real-time quantitative PCR was performed using the CFX96 real-time detection system (Bio-Rad Laboratories, Hercules, CA). Primers were synthesized by IDT, Inc. (Coralville, IA). The primer sequences are listed in Table 1 below. Accumulation of PCR products was detected by monitoring the increase in fluorescence of double-stranded DNA-binding dye SYBR Green during amplification. Relative gene expression was calculated by the comparative cycle threshold (Ct) method. The expression level of target genes was normalized to the house-keeping gene, 18S rRNA, in each sample and the fold-change in the target gene expression between experimental groups was expressed as a ratio. Melt-curve analysis was used to confirm the authenticity of the PCR products.

### miRNA Analysis

Tissue samples were lysed in QIAzol Lysis reagent (Qiagen, Maryland, USA), homogenized with stainless steel beads in TissueLyser II (Qiagen, Maryland, USA) and incubated on ice for five minutes followed by miRNA isolation using Direct-zol RNA MiniPrep kit with on column DNase digestion (Zymo Research, Irvin, CA). Reverse transcription (30 min −16 °C; 30 min −42 °C; 5 min −85 °C) was performed in Eppendorf Mastercycler (Eppendorf, New York, USA) using 10 ng RNA, TaqMan primers and MiRNA Reverse Transcription Kit (Applied Biosystems, Foster City, CA), followed by quantitative RT-PCR (10 min −95 °C; 40 cycles of 15 sec −95 °C; 1 min −60 °C) in CFX96 (Bio-Rad Laboratories, Hercules, CA) using TaqMan Universal PCR Master Mix (Biorad, Hercules, CA). All tissue results were normalized to snoRNA202 expression. Serum sample controls were spiked with *Caenorhabditis elegans* (cel)-miR-39, as per instructions (Qiagen, Gaithersburg, MD) and subsequently analyzed utilizing a primer pool. Briefly, serum cDNA synthesis was performed with a final 0.1x primer concentration and Applied Biosystems’s rtPCR kit followed by individual target-specific RT-qPCR analysis using Bio-Rad iTaq Universal Probes Master Mix according to manufacturer instructions.

### Western blot analysis

Whole cell lysates, nuclear and cytoplasmic extracts were prepared from mouse livers as described previously^60^. Proteins of interest were detected by immunoblotting with specific primary antibodies against: cyclin D1 (SC-753; santacurz), p53 (ab28; abcam), vimentin (ab92547; Abcam), Shh (SC-9024, Santa Cruz), β-tubulin-HRP (ab185057; Abcam), GAPDH-HRP (ab9482; Abcam). Respective horseradish peroxidase–labeled secondary antibodies were from Santacruz Biotechnology (Dallas, TX). The specific immunoreactive bands of interest were detected by chemiluminescence (Bio-Rad, Hercules, CA). The immunoreactive bands were quantified by densitometric analysis using the UVP System (Bio-Rad Laboratories, Hercules, CA).

### Histopathological analysis

Sections of formalin-fixed, paraffin-embedded livers were stained with hematoxylin and eosin (H&E), or Sirius Red and assessed for histological features of carcinoma and fibrosis. The H&E stained sections were independently examined by a veterinary pathologist, Dr. Garlick in a blinded manner (see acknowledgments). The quantitation of Sirius Red staining was performed using ImageJ software. Immunohistochemistry staining for AFP (ab46799; Abcam), CK7 (ab9021; Abcam), CK19 (ab52625; Abcam), was performed on formalin-fixed, paraffin-embedded livers according to the manufacturer’s instructions. To examine cell proliferation, mice were injected i.p. with 100 mg/kg BrdU (Sigma, St. Louise, MO) 2 hr prior to sacrifice, and paraffin sections were stained using the anti BrdU antibody (ab6326, Abcam). ImageJ (NIH) was used for image analysis.

### Statistical Analysis

Statistical significance was determined using two – tailed t-test; two-way ANOVA and Dunnett’s multiple comparison post-test were used to compare the means of multiple groups. Data are shown as mean ± SD and were considered statistically significant at p < 0.05. GraphPad Prism 6.02 (GraphPad Software Inc., La Jolla, CA) was used for analysis.

## Additional Information

**How to cite this article**: Ambade, A. *et al*. Alcoholic hepatitis accelerates early hepatobiliary cancer by increasing stemness and miR-122-mediated HIF-1α activation. *Sci. Rep*. **6**, 21340; doi: 10.1038/srep21340 (2016).

## Supplementary Material

Supplementary Information

## Figures and Tables

**Table 1:** Primers used in this study.

Gene	Forward Primer – 5′-3′	Reverse primer – 5′-3′
Gli1	GTCACTACCTGGCCTCACAC	AAGACCTCCCATCCGATCCA
CCND2	AGGCTGTTTCTGTGGTCTCG	GCTGCTCCCGTTTTCTCTCT
E-cadherin	GGTATCTTGGTGTGGGTGCA	AATCTGAGACGTGTGCAGCA
N-cadherin	CACTGCCATTGATGCGGATG	TGCCACAGTGATGATGTCCC
Nanog	CTGGGCTTAAAGTCAGGGCA	AAGATCTGACGCCCTCCTCT
Vimentin	CGG AAA GTG GAA TCC TTG CA	CAC ATC GAT CTG GAC ATG CTG T
Bcl-w	AACTCACAGTCCAGTCCCCT	GAAGTGCAGCAGTGAGGTCT
CCNG1	ACT CGT TCA CGA CAC CTT GCC A	GCC AGC ACA GAA GGC TTT GCC
TNFα	CAC CAC CAT CAA GGA CTC AA	AGG CAA CCT GAC CAC TCT CC
IL-6	ACA ACC ACG GCC TTC CCT ACT T	CAC GAT TTC CCA GAG AAC ATG TG
MCP-1	CAG GTC CCT GTC ATG CTT CT	CAG GTC CCT GTC ATG CTT CT
IL-17A	TCC CTC TGT GAT CTG GGA AG	CTC GAC CCT GAA AGT GAA GG
αSMA	GTC CCA GAC ATC AGG GAG TAA	TCG GAT ACT TCA GCG TCA GGA
TGFβ	ATT CCT GGC GTT ACC TTG	CTG TAT TCC GTC TCC TTG GTT
Procollagen	GCT CCT CTT AGG GGC CAC T	CCA CGT CTC ACC ATT GGG G
AFP	CTCCGAGTCCAGAAGGAAGAGTGGAC	GCGGCCGCAGACTAGGAGAAGAGAAATAGTT
CCND1	AGC CTC CAG AGG GCT GTC GG	TGG GGA GGG CTG TGG TCT CG
p53	CAC GTA CTC TCC TCC CCT CAA T	AAC TGC ACA GGG CAC GTC TT
CD133	GAA AAG TTG CTC TGC GAA CC	TCT CAA GCT GAA AAG CAG CA
OPN	CTTTCACTCCAATCGTCCCTAC	GGTCCTCATCTGTGGCATCA
CD44	CACCATCGAGAAGAGCACCC	GAATGACTCTGTGTGGTGGC
HIF1α	CAA GAT CTC GGC GAA GCA A	GGT GAG CCT CAT AAC AGA AGC TTT
VEGFR1	ACA TTG GTG GTG GCT GAC TCT C	CCT CTC CTT CGG CTG GCA TC
p21	TAG GGG AAT TGG AGT CAG GC	AGA GAC AAC GGC ACA CTT TG
18 s	GTA ACC CGT TGA ACC CCA TT	CCA TCC AAT CGG TAG TAG CG

## References

[b1] El-Serag H. B. Hepatocellular carcinoma. N. Engl. J. Med. 365, 1118–1127 (2011). 21992124 10.1056/NEJMra1001683

[b2] Torre L. A. . Global cancer statistics, 2012. CA Cancer. J. Clin. 65, 87–108 (2015). 25651787 10.3322/caac.21262

[b3] Cancer Facts and Figures 2015. American Cancer Society, Atlanta. (2015).

[b4] Ferlay J. . Cancer incidence and mortality worldwide: sources, methods and major patterns in GLOBOCAN 2012. Int. J. Cancer 136, E359–86 (2015). 25220842 10.1002/ijc.29210

[b5] Altekruse S. F., Henley S. J., Cucinelli J. E. & McGlynn K. A. Changing hepatocellular carcinoma incidence and liver cancer mortality rates in the United States. Am. J. Gastroenterol. 109, 542–553 (2014). 24513805 10.1038/ajg.2014.11 PMC4148914

[b6] Sanyal A. J., Yoon S. K. & Lencioni R. The etiology of hepatocellular carcinoma and consequences for treatment. Oncologist 15 Suppl 4, 14–22 (2010). 21115577 10.1634/theoncologist.2010-S4-14

[b7] Villanueva A., Newell P., Chiang D. Y., Friedman S. L. & Llovet J. M. Genomics and signaling pathways in hepatocellular carcinoma. Semin. Liver Dis. 27, 55–76 (2007). 17295177 10.1055/s-2006-960171

[b8] Heindryckx F., Colle I. & Van Vlierberghe H. Experimental mouse models for hepatocellular carcinoma research. Int. J. Exp. Pathol. 90, 367–386 (2009). 19659896 10.1111/j.1365-2613.2009.00656.x PMC2741148

[b9] Verna L., Whysner J. & Williams G. M. N-nitrosodiethylamine mechanistic data and risk assessment: bioactivation, DNA-adduct formation, mutagenicity and tumor initiation. Pharmacol. Ther. 71, 57–81 (1996). 8910949 10.1016/0163-7258(96)00062-9

[b10] Brandon-Warner E., Walling T. L., Schrum L. W. & McKillop I. H. Chronic ethanol feeding accelerates hepatocellular carcinoma progression in a sex-dependent manner in a mouse model of hepatocarcinogenesis. Alcohol. Clin. Exp. Res. 36, 641–653 (2012). 22017344 10.1111/j.1530-0277.2011.01660.x PMC3288433

[b11] Cook R. T. . Thymocytes, pre-B cells and organ changes in a mouse model of chronic ethanol ingestion—absence of subset-specific glucocorticoid-induced immune cell loss. Alcohol. Clin. Exp. Res. 31, 1746–1758 (2007). 17681030 10.1111/j.1530-0277.2007.00478.x PMC2190628

[b12] Elinav E. . Inflammation-induced cancer: crosstalk between tumours, immune cells and microorganisms. Nat. Rev. Cancer. 13, 759–771 (2013). 24154716 10.1038/nrc3611

[b13] Kitisin K., Pishvaian M. J., Johnson L. B. & Mishra L. Liver stem cells and molecular signaling pathways in hepatocellular carcinoma. Gastrointest. Cancer. Res. 1, S13–21 (2007). 19360142 PMC2666844

[b14] Szabo G. Gut-Liver Axis in Alcoholic Liver Disease. Gastroenterology 148, 30–36 (2015). 25447847 10.1053/j.gastro.2014.10.042 PMC4274189

[b15] Ma S. . IL-17A produced by gammadelta T cells promotes tumor growth in hepatocellular carcinoma. Cancer Res. 74, 1969–1982 (2014). 24525743 10.1158/0008-5472.CAN-13-2534

[b16] Frazier T. H., Stocker A. M., Kershner N. A., Marsano L. S. & McClain C. J. Treatment of alcoholic liver disease. Therap Adv. Gastroenterol. 4, 63–81 (2011). 10.1177/1756283X10378925 PMC3036962 21317995

[b17] Tang K. H. . CD133(+) liver tumor-initiating cells promote tumor angiogenesis, growth and self-renewal through neurotensin/interleukin-8/CXCL1 signaling. Hepatology 55, 807–820 (2012). 21994122 10.1002/hep.24739

[b18] Machida K. . Toll-like receptor 4 mediates synergism between alcohol and HCV in hepatic oncogenesis involving stem cell marker Nanog. Proc. Natl. Acad. Sci. USA 106, 1548–1553 (2009). 19171902 10.1073/pnas.0807390106 PMC2635765

[b19] Abelev G. I. & Eraiser T. L. Cellular aspects of alpha-fetoprotein reexpression in tumors. Semin. Cancer Biol. 9, 95–107 (1999). 10202131 10.1006/scbi.1998.0084

[b20] Lowes K. N., Brennan B. A., Yeoh G. C. & Olynyk J. K. Oval cell numbers in human chronic liver diseases are directly related to disease severity. Am. J. Pathol. 154, 537–541 (1999). 10027411 10.1016/S0002-9440(10)65299-6 PMC1849988

[b21] Chen Y., Wong P. P., Sjeklocha L., Steer C. J. & Sahin M. B. Mature hepatocytes exhibit unexpected plasticity by direct dedifferentiation into liver progenitor cells in culture. Hepatology 55, 563–574 (2012). 21953633 10.1002/hep.24712 PMC3268884

[b22] Sell S. & Leffert H. L. Liver cancer stem cells. J. Clin. Oncol. 26, 2800–2805 (2008). 18539957 10.1200/JCO.2007.15.5945 PMC2515096

[b23] Fausto N. & Campbell J. S. The role of hepatocytes and oval cells in liver regeneration and repopulation. Mech. Dev. 120, 117–130 (2003). 12490302 10.1016/s0925-4773(02)00338-6

[b24] Joo M., Kang Y. K., Kim M. R., Lee H. K. & Jang J. J. Cyclin D1 overexpression in hepatocellular carcinoma. Liver 21, 89–95 (2001). 11318977 10.1034/j.1600-0676.2001.021002089.x

[b25] van Zijl F. . Epithelial-mesenchymal transition in hepatocellular carcinoma. Future Oncol. 5, 1169–1179 (2009). 19852728 10.2217/fon.09.91 PMC2963061

[b26] Nakagawa H. . Loss of liver E-cadherin induces sclerosing cholangitis and promotes carcinogenesis. Proc. Natl. Acad. Sci. USA 111, 1090–1095 (2014). 24395807 10.1073/pnas.1322731111 PMC3903249

[b27] Katoh Y. & Katoh M. Hedgehog signaling pathway and gastrointestinal stem cell signaling network (review). Int. J. Mol. Med. 18, 1019–1023 (2006). 17089004

[b28] Callegari E. . MicroRNAs in liver cancer: a model for investigating pathogenesis and novel therapeutic approaches. Cell Death Differ. 22, 46–57 (2015). 25190143 10.1038/cdd.2014.136 PMC4262781

[b29] Coulouarn C., Factor V. M., Andersen J. B., Durkin M. E. & Thorgeirsson S. S. Loss of miR-122 expression in liver cancer correlates with suppression of the hepatic phenotype and gain of metastatic properties. Oncogene 28, 3526–3536 (2009). 19617899 10.1038/onc.2009.211 PMC3492882

[b30] Gramantieri L. . Cyclin G1 is a target of miR-122a, a microRNA frequently down-regulated in human hepatocellular carcinoma. Cancer Res. 67, 6092–6099 (2007). 17616664 10.1158/0008-5472.CAN-06-4607

[b31] Fornari F. . MiR-122/cyclin G1 interaction modulates p53 activity and affects doxorubicin sensitivity of human hepatocarcinoma cells. Cancer Res. 69, 5761–5767 (2009). 19584283 10.1158/0008-5472.CAN-08-4797

[b32] Rockwell S., Dobrucki I. T., Kim E. Y., Marrison S. T. & Vu V. T. Hypoxia and radiation therapy: past history, ongoing research and future promise. Curr. Mol. Med. 9, 442–458 (2009). 19519402 10.2174/156652409788167087 PMC2752413

[b33] Wu X. Z., Xie G. R. & Chen D. Hypoxia and hepatocellular carcinoma: The therapeutic target for hepatocellular carcinoma. J. Gastroenterol. Hepatol. 22, 1178–1182 (2007). 17559361 10.1111/j.1440-1746.2007.04997.x

[b34] Csak T. . microRNA-122 regulates hypoxia-inducible factor-1 and vimentin in hepatocytes and correlates with fibrosis in diet-induced steatohepatitis. Liver Int. 35, 532–541 (2015). 25040043 10.1111/liv.12633 PMC4289469

[b35] Wang J., Zhang K. Y., Liu S. M. & Sen S. Tumor-associated circulating microRNAs as biomarkers of cancer. Molecules 19, 1912–1938 (2014). 24518808 10.3390/molecules19021912 PMC6271223

[b36] Koberle V. . Serum microRNA-1 and microRNA-122 are prognostic markers in patients with hepatocellular carcinoma. Eur. J. Cancer 49, 3442–3449 (2013). 23810247 10.1016/j.ejca.2013.06.002

[b37] O’Shea R. S., Dasarathy S. & McCullough A. J., Practice Guideline Committee of the American Association for the Study of Liver Diseases & Practice Parameters Committee of the American College of Gastroenterology. Alcoholic liver disease. Hepatology 51, 307–328 (2010). 20034030 10.1002/hep.23258

[b38] Brandon-Warner E., Schrum L. W., Schmidt C. M. & McKillop I. H. Rodent models of alcoholic liver disease: of mice and men. Alcohol 46, 715–725 (2012). 22960051 10.1016/j.alcohol.2012.08.004 PMC3496818

[b39] Lieber C. S. & DeCarli L. M. The feeding of alcohol in liquid diets: two decades of applications and 1982 update. Alcohol. Clin. Exp. Res. 6, 523–531 (1982). 6758624 10.1111/j.1530-0277.1982.tb05017.x

[b40] Chowdhury G., Calcutt M. W., Nagy L. D. & Guengerich F. P. Oxidation of methyl and ethyl nitrosamines by cytochrome P450 2E1 and 2B1. Biochemistry 51, 9995–10007 (2012). 23186213 10.1021/bi301092c PMC3525961

[b41] Waris G. & Ahsan H. Reactive oxygen species: role in the development of cancer and various chronic conditions. J. Carcinog. 5, 14 (2006). 16689993 10.1186/1477-3163-5-14 PMC1479806

[b42] Reuter S., Gupta S. C., Chaturvedi M. M. & Aggarwal B. B. Oxidative stress, inflammation and cancer: how are they linked? Free Radic. Biol. Med. 49, 1603–1616 (2010). 20840865 10.1016/j.freeradbiomed.2010.09.006 PMC2990475

[b43] Umemura T. . Pentachlorophenol (but not phenobarbital) promotes intrahepatic biliary cysts induced by diethylnitrosamine to cholangio cystic neoplasms in B6C3F1 mice possibly due to oxidative stress. Toxicol. Pathol. 31, 10–13 (2003). 12597444 10.1080/01926230390173806

[b44] Soreide K. & Soreide J. A. Bile duct cyst as precursor to biliary tract cancer. Ann. Surg. Oncol. 14, 1200–1211 (2007). 17187167 10.1245/s10434-006-9294-3

[b45] Tyson G. L. & El-Serag H. B. Risk factors for cholangiocarcinoma. Hepatology 54, 173–184 (2011). 21488076 10.1002/hep.24351 PMC3125451

[b46] Welzel T. M. . Risk factors for intrahepatic and extrahepatic cholangiocarcinoma in the United States: a population-based case-control study. Clin. Gastroenterol. Hepatol. 5, 1221–1228 (2007). 17689296 10.1016/j.cgh.2007.05.020 PMC2083573

[b47] Yamashita T. & Wang X. W. Cancer stem cells in the development of liver cancer. J. Clin. Invest. 123, 1911–1918 (2013). 23635789 10.1172/JCI66024 PMC3635728

[b48] Oishi N., Yamashita T. & Kaneko S. Molecular biology of liver cancer stem cells. Liver Cancer. 3, 71–84 (2014). 24944998 10.1159/000343863 PMC4057789

[b49] Rountree C. B. . A CD133-expressing murine liver oval cell population with bilineage potential. Stem Cells 25, 2419–2429 (2007). 17585168 10.1634/stemcells.2007-0176

[b50] Shuang Z. Y. . Transforming growth factor-beta1-induced epithelial-mesenchymal transition generates ALDH-positive cells with stem cell properties in cholangiocarcinoma. Cancer Lett. 354, 320–328 (2014). 25194504 10.1016/j.canlet.2014.08.030

[b51] Philips G. M. . Hedgehog signaling antagonist promotes regression of both liver fibrosis and hepatocellular carcinoma in a murine model of primary liver cancer. PLoS One 6, e23943 (2011). 21912653 10.1371/journal.pone.0023943 PMC3166282

[b52] Razumilava N. . Non-canonical Hedgehog signaling contributes to chemotaxis in cholangiocarcinoma. J. Hepatol. 60, 599–605 (2014). 24239776 10.1016/j.jhep.2013.11.005 PMC3944428

[b53] Tsai W. C. . MicroRNA-122 plays a critical role in liver homeostasis and hepatocarcinogenesis. J. Clin. Invest. 122, 2884–2897 (2012). 22820290 10.1172/JCI63455 PMC3408747

[b54] Kutay H. . Downregulation of miR-122 in the rodent and human hepatocellular carcinomas. J. Cell. Biochem. 99, 671–678 (2006). 16924677 10.1002/jcb.20982 PMC3033198

[b55] Hsu S. H. . Essential metabolic, anti-inflammatory and anti-tumorigenic functions of miR-122 in liver. J. Clin. Invest. 122, 2871–2883 (2012). 22820288 10.1172/JCI63539 PMC3408748

[b56] Bai S. . MicroRNA-122 inhibits tumorigenic properties of hepatocellular carcinoma cells and sensitizes these cells to sorafenib. J. Biol. Chem. 284, 32015–32027 (2009). 19726678 10.1074/jbc.M109.016774 PMC2797273

[b57] Thakral S. & Ghoshal K. miR-122 is a Unique Molecule with Great Potential in Diagnosis, Prognosis of Liver Disease and Therapy Both as miRNA Mimic and Antimir. Curr. Gene Ther. 15, 142–150 (2015). 25537773 10.2174/1566523214666141224095610 PMC4439190

[b58] Nakao K., Miyaaki H. & Ichikawa T. Antitumor function of microRNA-122 against hepatocellular carcinoma. J. Gastroenterol. 49, 589–593 (2014). 24531873 10.1007/s00535-014-0932-4

[b59] Semenza G. L. HIF-1 mediates metabolic responses to intratumoral hypoxia and oncogenic mutations. J. Clin. Invest. 123, 3664–3671 (2013). 23999440 10.1172/JCI67230 PMC3754249

[b60] Nath B. . Hepatocyte-specific hypoxia-inducible factor-1alpha is a determinant of lipid accumulation and liver injury in alcohol-induced steatosis in mice. Hepatology 53, 1526–1537 (2011). 21520168 10.1002/hep.24256 PMC3104403

[b61] Pirola C. J. . Circulating microRNA signature in non-alcoholic fatty liver disease: from serum non-coding RNAs to liver histology and disease pathogenesis. Gut 64, 800–812 (2015). 24973316 10.1136/gutjnl-2014-306996 PMC4277726

[b62] Bala S. . Circulating microRNAs in exosomes indicate hepatocyte injury and inflammation in alcoholic, drug-induced and inflammatory liver diseases. Hepatology 56, 1946–1957 (2012). 22684891 10.1002/hep.25873 PMC3486954

[b63] Shifeng H. . Circulating liver-specific miR-122 as a novel potential biomarker for diagnosis of cholestatic liver injury. PLoS One 8, e73133 (2013). 24086271 10.1371/journal.pone.0073133 PMC3785475

[b64] Xu J. . Circulating microRNAs, miR-21, miR-122 and miR-223, in patients with hepatocellular carcinoma or chronic hepatitis. Mol. Carcinog. 50, 136–142 (2011). 21229610 10.1002/mc.20712

