# Supplementary material for: Alcoholic hepatitis accelerates early hepatobiliary cancer by increasing stemness and miR-122-mediated HIF-1α activation
Source: Sci Rep. 2016 Feb 18;6:21340. doi: 10.1038/srep21340 (PMC4758032; doi:10.1038/srep21340)
Supplement: Supplementary Information [file srep21340-s1.pdf]

Supplementary Information

Alcoholic hepatitis accelerates early hepatobiliary cancer by increasing stemness and miR-122-mediated HIF-1α activation

Aditya Ambade, Abhishek Satishchandran, Gyongyi Szabo*

Department of Medicine, University of Massachusetts Medical School, Worcester, MA 01604. United States.

Supplementary Figure 1

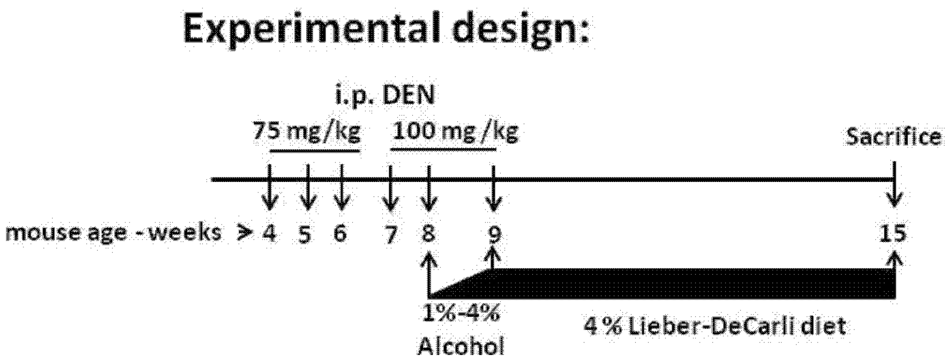

Supplementary Figure S1. Experimental design of alcohol accelerated model of hepatobiliary neoplasia (n ≥ 5 mice per group).

Supplementary Figure 2

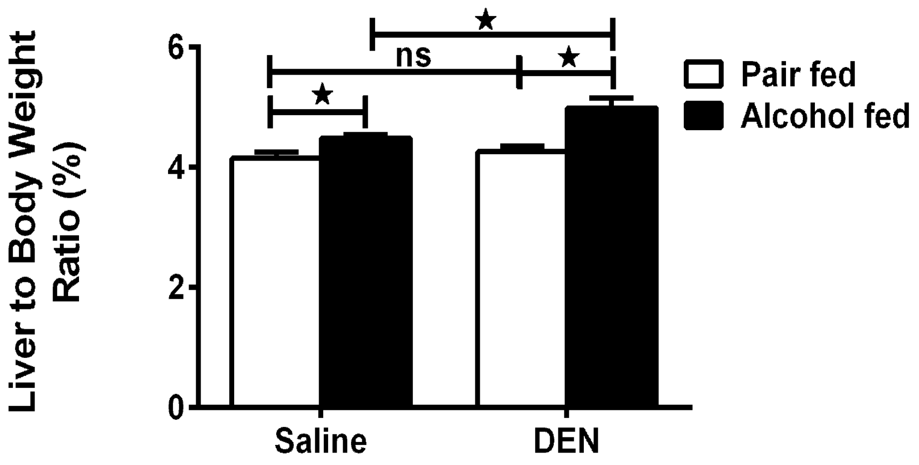

Supplementary figure S2. Liver to body weight ratio at the time of sacrifice. In the graph, values are given as average ± SD, ANOVA and Dunnett's multiple comparison were used to compare the means of multiple groups; (*$p<0.05$).
